# Cu-Related Paramagnetic Centers in Cu- and (Cu,Y)-Doped ZrO_2_ Nanopowders

**DOI:** 10.3390/ma18030605

**Published:** 2025-01-29

**Authors:** Valentyna Nosenko, Igor Vorona, Volodymyr Trachevsky, Yuriy Zagorodniy, Sergey Okulov, Oksana Isaieva, Volodymyr Yukhymchuk, Sergei A. Kulinich, Lyudmyla Borkovska, Larysa Khomenkova

**Affiliations:** 1 V. Lashkaryov Institute of Semiconductor Physics, National Academy of Sciences of Ukraine, 45 Pr. Nauky, 03028 Kyiv, Ukraine; 2 Faculty of Natural Sciences, National University “Kyiv-Mohyla Academy”, 2 Skovorody Str., 04070 Kyiv, Ukraine; 3 Technical Center, National Academy of Sciences of Ukraine, 13 Pokrovs’ka Str., 04070 Kyiv, Ukraine; 4 Frantsevych Institute for Problems of Materials Science, National Academy of Sciences of Ukraine, 3 O. Pritsaka Str., 03142 Kyiv, Ukraine; 5 Research Institute of Science and Technology, Tokai University, Hiratsuka, Kanagawa 259-1292, Japan

**Keywords:** zirconia, doping, copper, EPR, NMR, Raman, paramagnetic centers

## Abstract

In this work, we studied Cu-doped and (Cu,Y)-codoped ZrO_2_ nanopowders produced through a coprecipitation approach to identify the nature of Cu-related bulk and surface paramagnetic centers. We conducted EPR, NMR, and Raman scattering studies on Cu- and (Cu,Y)-doped ZrO_2_ powders calcined at different temperatures. At low calcination temperatures (400 °C) and low Cu loading (0.1–1.0 mol.% of CuO), the EPR signal was found to be attributed to surface-related Cu-H_2_O complexes. For powders with higher Cu content (up to 8.0 mol.% of CuO), the superparamagnetic signal associated with the formation of copper clusters was observed. At higher calcination temperatures, the destruction of Cu-related surface complexes promotes the incorporation of Cu^2+^ ions into the bulk of ZrO_2_ nanocrystals at Zr positions. Co-doping ZrO_2_ with Cu and Y was observed to facilitate the incorporation of Cu^2+^ ions into cation sites at lower calcination temperatures when compared with Cu-doped ZrO_2_.

## 1. Introduction

Zirconium dioxide (ZrO_2_), or zirconia, exhibits several remarkable properties, including high thermal resistance, hardness, transparency, a high refractive index, chemical stability, and a high melting point. These properties have led to its use in various applications such as coatings, catalysis, and even sensing [1,2,3,4,5,6,7,8,9,10,11,12,13]. Doping zirconium dioxide with various impurities can impart new properties, expanding its range of applications. For example, zirconia ceramics doped with rare earth elements exhibit a wide range of luminescent features, making them suitable for use in light-emitting devices [1,2]. Additionally, doping ZrO_2_ with copper enhances its catalytic properties, enabling the production of highly efficient catalysts [5,6,7,8].

The incorporation of sub-valent impurities into the structure of zirconium dioxide is a complex and poorly understood process due to two main reasons. The first reason is that the doping impurity, depending on the technology used for its introduction, can occupy different structural positions. It can either enter directly into the zirconium dioxide structure or localize on the surface of granules, thus forming various surface complexes. The second reason is that the inclusion of sub-valent impurities in the zirconium dioxide structure requires charge compensation, which can lead to significant restructuring of the oxide itself. Pure ZrO_2_ crystals typically have a monoclinic structure at room temperature. Upon heating to around 1460 °C, it transforms into the tetragonal phase and then into the cubic phase at higher temperatures. However, it becomes unstable upon cooling, undergoing reverse transformations from cubic to tetragonal to monoclinic phases.

For ZrO_2_ powders, the same processes are observed, with the difference that phase transitions occur at lower temperatures and depend on the size of the powder granules. An exception is powdered with nanosized granules, where the stabilization of high-temperature phases at room temperature is possible due to surface energy. The introduction of sub-valent impurities, such as the frequently used yttrium, into the structure of zirconium dioxide stabilizes the tetragonal or cubic ZrO_2_ phases at room temperature, depending on the concentration of the impurities [14,15,16]. The stabilization of both phases is believed to be due to the formation of oxygen vacancies required for dopant charge compensation [4,17]. Therefore, knowledge of the localization of sub-valent impurities and the identification of associated defects is important for understanding the properties of doped zirconium dioxide. The use of the electron paramagnetic resonance method for this purpose has proven to be a powerful tool in the study of intrinsic and impurity defects in various solid-state materials.

As mentioned above, copper doping of zirconium dioxide is used to enhance its catalytic properties. This enhancement is believed to be due to the formation of complexes containing copper, oxygen, and hydrogen atoms on the surface of zirconium dioxide powder granules. Previous studies have shown that when zirconium dioxide powders and ceramics are doped with copper, the copper atoms can be incorporated into the ZrO_2_ structure, as well as form various complexes on the surface. For example, paramagnetic centers Cu_Zr_^2+^ have been detected in yttrium-stabilized ZrO_2_ ceramics, along with aggregates of Cu atoms, CuO molecules, and crystalline CuO located on the surface of granules and/or between powder grains [9,11,18,19]. However, up to date, these studies were not systematic.

Therefore, the present work is devoted to the study of electron paramagnetic resonance (EPR) of paramagnetic centers associated with copper in zirconium oxide powders calcined at different temperatures. The dependence of the formation of these paramagnetic centers on the concentration of introduced copper and the effect of co-doping with yttrium were also investigated. To clarify the nature and/or model of paramagnetic centers, nuclear magnetic resonance (NMR) and Raman spectroscopy techniques were additionally used.

The identification of paramagnetic centers observed in the EPR spectrum will facilitate the development of a fast and non-destructive method to control Cu-doped ZrO_2_ materials. Additionally, this will open up opportunities to optimize technological parameters for creating effective catalysts, using only a small amount of the investigated powders required for EPR research.

## 2. Materials and Methods

A co-precipitation technique was applied to synthesize Cu-doped and (Cu,Y)-codoped ZrO_2_ powders from ZrO(NO_3_)_2_·nH_2_O, Y(NO_3_)_3_, and Cu(NO_3_)_2_ precursors. The Zr and Cu salts and Zr, Cu, and Y salts were taken in the required ratio to produce Cu-doped and (Cu,Y)-codoped ZrO_2_ powders, respectively. The composition of the prepared powders is given in Table 1.

More details about the preparation of the powders studied here can be found elsewhere [4]. When the co-precipitation process was completed, the gel-like substance was dried at 80 °C for 48 h to remove water molecules and subsequently heated at 150 °C for 24 h to complete the process. The resulting sediments underwent further calcination at 400, 600, 800, and 1000 °C for 2 h in air and then slowly cooled in the furnace to room temperature. As a result, nano-sized powders with average particle sizes ranging from 8 to 15 nm were obtained, depending on the calcination temperature. More details on their structural characterization can be found in ref. [4].

Electron paramagnetic resonance (EPR) measurements were carried out using an X-band Varian E12 (Varian, Palo Alto, CA, USA) and Bruker EPR ELEXSYS 580 (Bruker Corporation, Billerica, MA, USA) spectrometers at room temperature and at liquid nitrogen temperature. The powders studied were placed in a quartz tube with an internal diameter of 4 mm, where their EPR signals were recorded with unsaturated microwave power (2 mW). A 100 kHz modulation of the magnetic field with peak-to-peak amplitude modulation of 0.1 mT was applied. The signal of an MgO:Mn sample containing 3 × 10^15^ spins was used as a reference. The obtained EPR spectra were normalized to the intensity of the MgO:Mn reference signal, taking into account the mass of each studied powder. Spectra simulations were performed using the WINEPR Simfonia software package (Version 1.25).

Magic-angle spinning nuclear magnetic resonance (MAS NMR) spectra of powdered samples were recorded on a 400 MHz commercial Bruker Avance NMR spectrometer (Bruker Corporation, USA) in a magnetic field of 9.40 T, at room temperature and with a spinning frequency of 5 kHz. ^1^H MAS NMR spectra were acquired using a single pulse sequence with a recycle delay of 5 s. The spectra were referenced against the TMS, which was taken here as the zero for the chemical shift.

The powders were also studied by means of the Raman scattering method. To record the Raman spectra, an MDR-23 spectrometer (LOMO, St. Petersburg, Russia) equipped with a cooled CCD detector, iDus 420 Andor (London, UK), was used. A diode-pumped solid-state 457 nm laser was used as the excitation source. The laser power density on the sample surface was less than 10^3^ W/cm^2^ to prevent structural transformation due to laser heating. Spectral calibration was performed using the peak position of a 520.6 cm^−1^ phonon peak of a silicon single crystal, with a peak width of approximately 4 cm^−1^.

## 3. Results and Discussion

**Figure 1:**
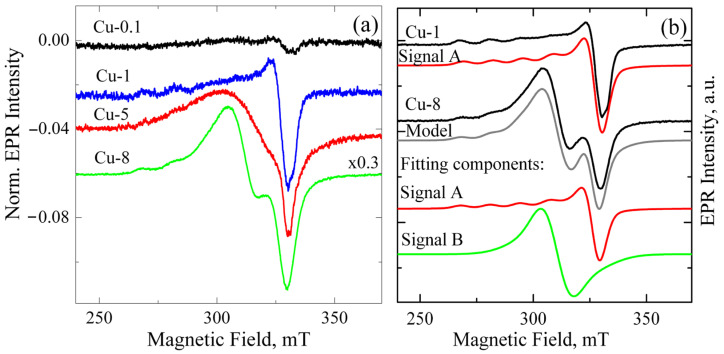
(**a**) EPR spectra of Cu-doped ZrO_2_ powders calcined at 400 °C. The Cu content varies from 0.1 to 8 mol.%. Spectra are normalized by powder mass and to the amplitude of the reference sample; (**b**) experimental and model EPR spectra of Cu-1 and Cu-8 powders calcined at 400 °C. Fitting components for each sample are also shown as red and green curves.

Figure 1a shows the EPR signals from ZrO_2_ samples alloyed with different Cu contents ranging from 0.1 to 8 mol.% and calcined at 400 °C. The spectra were normalized by mass and the intensity of the reference sample, permitting direct comparison of signal intensity across different samples. The total intensity of the EPR signal shows a direct correlation with the increase in Cu loading, as illustrated in Figure 1a. This indicates that the observed EPR signal is caused by either copper ions themselves or by the associated defects formed when copper enters the powders.

In parallel, changes observed in the shape of the spectra indicate the appearance of an additional signal. A detailed analysis of the EPR spectra shows that the EPR spectrum of ZrO_2_ powder doped with up to 1 mol.% CuO can be described using a single-curve model, denoted as signal A (see Figure 1b). Such spectra exhibit the characteristic for Cu^2+^ (3d^9^) species hyperfine splitting into four lines, well resolved in the g_||_ region, due to the hyperfine coupling between unpaired electron (S = 1/2) and Cu nucleus (I = 3/2). Hyperfine splitting A_⊥_ is usually very small, and due to its line width, the spectrum shows no splitting at the g_⊥_ region. This spectrum reveals that the Cu^2+^ center is axial (g_||_ > g_⊥_ > g_e_) and can be described by corresponding parameters g_⊥_ = 2.063, g_||_ = 2.340, and A_||_ = 130 G. Its parameters are intermediate between Cu(OH)_4_^2−^ with g_||_ = 2.29 and Cu(H_2_O)_4_^2+^ with g_||_ = 2.42 [20]. The highest concentration of these paramagnetic centers was observed in sample Cu-1, at 2 × 10^17^ spins/cm^3^.

To describe the EPR spectra of ZrO_2_ samples doped with 5 mol.% and 8 mol.% CuO, an additional component with g ~ 2.17 (Signal B) was required. Its shape and position were somewhat different for ZrO_2_ samples doped with 5 and 8 mol.% CuO. Since the intensity of signal B increases with rising Cu content, it can be assumed that this signal relates to copper-enriched areas, where adjacent Cu^2+^ ions are coupled by strong dipole–dipole or exchange interactions. These may be heavily doped near-surface regions of granules, metallic copper, or/and CuO_x_ nanoclusters on the grain surface. Previously, copper clusters were reported to exhibit superparamagnetic behavior [21].

**Figure 2:**
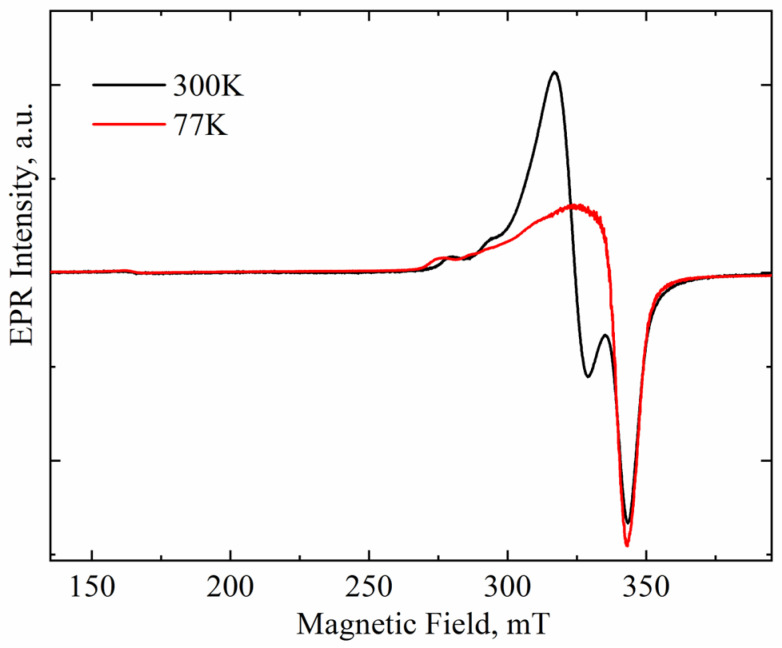
Spectra of Cu-doped ZrO_2_ powders (Cu-8) calcined at 400 °C and recorded at room and nitrogen temperature. Spectra normalized on Fe^3+^ signal intensity.

The intensity of paramagnetic signals changes with the measurement temperature in accordance with the Curie–Weiss law. At the same time, the intensity of the superparamagnetic signal depends weakly on temperature. Therefore, comparison of EPR spectra of a sample recorded at different temperatures allows one to separate the paramagnetic and superparamagnetic components in the spectra. Figure 2 compares the EPR spectra of ZrO_2_ doped with 8 mol.% CuO recorded at 300 and 77 K. Both spectra are seen to exhibit a very weak signal at g ≈ 4, caused by an uncontrolled iron impurity in the quartz tube used to hold the studied powders. This signal is paramagnetic. Since it does not overlap with the signals from the ZrO_2_ powder, normalizing the experimental spectra is convenient to clarify the nature of the EPR signals studied.

The intensities of signals A and that of iron were found to change synchronously with the measurement temperature, thus following the Curie–Weiss law, which indicates the paramagnetic nature of signal A. At the same time, the intensity of signal B increased slightly at lower temperatures, thus indicating its superparamagnetic nature [22]. This finding allows for the unambiguous identification of signal B with copper-rich nanoclusters, such as metallic copper or CuO_x_ located on the surface of ZrO_2_ powder granules.

The nature of signal A cannot be determined based on EPR data alone. Similar EPR spectra have been observed in ZrO_2_ and other copper-doped oxides (TiO_2_, ZnO, etc.), which was attributed to copper ions in Cu-related surface complexes. In those complexes, copper ions were proposed to be situated in tetragonally distorted octahedral fields of ligands such as H_2_O, O^−^, SO_4_^2−^, and OH^−^ [23,24]. The nature of this signal will be discussed in more detail after presenting the NMR data.

**Figure 3:**
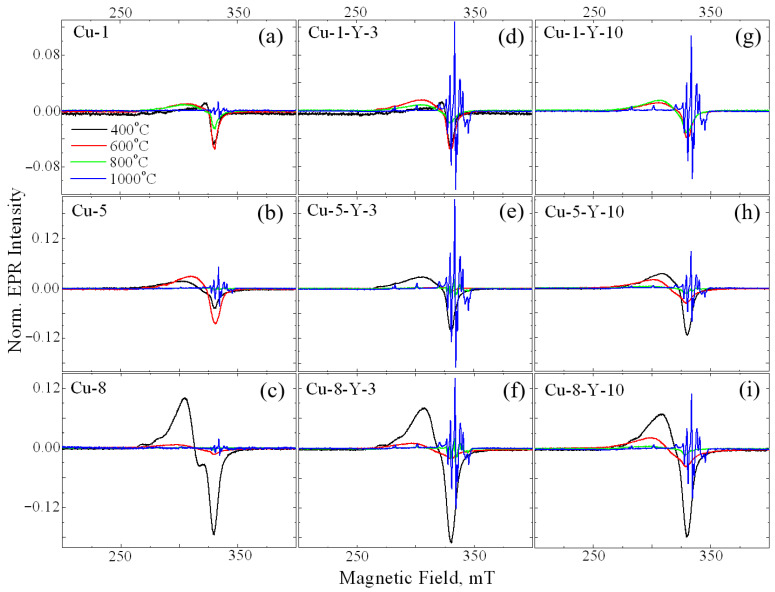
EPR spectra of Cu-doped (**a**–**c**) and (Cu,Y)-codoped (**d**–**i**) ZrO_2_ powders calcined at 400 (black line), 600 (red line), 800 (green line), and 1000 °C (blue line). The CuO loading is 1 mol.% (**a**,**d**,**g**), 5 mol.% (**b**,**e**,**h**) and 8 mol.% (**c**,**f**,**i**). The Y_2_O_3_ loading is 3 mol.% (**d**–**f**) and 10 mol.% (**g**–**i**).

Calcination temperature was found to drastically influence the line shape of the EPR signal of Cu-ZrO_2_ powders, as seen in Figure 3a–c. Signal A is seen to decrease with calcination temperature and almost disappears at 800 °C. In the samples calcined at 1000 °C (see Figure 3a–c, blue lines), a set of narrow lines of an irregular shape is observed in a wide range of magnetic fields. The small line width of this signal indicates a strictly regular crystallographic environment of the corresponding paramagnetic center, excluding the scattering of radio-spectroscopic parameters of the center. Previously, this signal was observed by Vorona and coworkers [19] and was assigned to Cu^2+^_Zr_ in the monoclinic ZrO_2_ lattice (denoted as Signal C). Its radio-spectroscopic parameters are S = 1/2, I = 3/2, g_x_ = 2.021, g_y_ = 2.026, g_z_ = 2.168, A_xx_ ~ 40 × 10^−4^ cm^−1^, A_yy_ ~ 35 × 10^−4^ cm^−1^, A_zz_ ~ 186 × 10^−4^ cm^−1^, A_xz_ ~ 10 × 10^−4^ cm^−1^, and A_yz_ ~ 5 × 10^−4^ cm^−1^ for the ^63^Cu isotope. This can be explained by the fact that part of the Cu^2+^ ions, which appeared as a result of the destruction of surface complexes, is additionally incorporated into the bulk of nanocrystals.

An increase in copper loading up to 1 mol% leads to an increase in the intensity of signal A. Further increases in the alloying impurity loading do not cause a noticeable increase in the intensity of signal A. Additionally, at 5 mol% CuO, signal B appears (Figure 3b), with its intensity increasing as copper loading increases up to 8 mol% (Figure 3c). An increase in the calcination temperature results in a decrease in the intensity of signal A. At a calcination temperature of 1000 °C, both signals A and B disappear, and only signal C is observed in the EPR spectrum.

**Figure 4:**
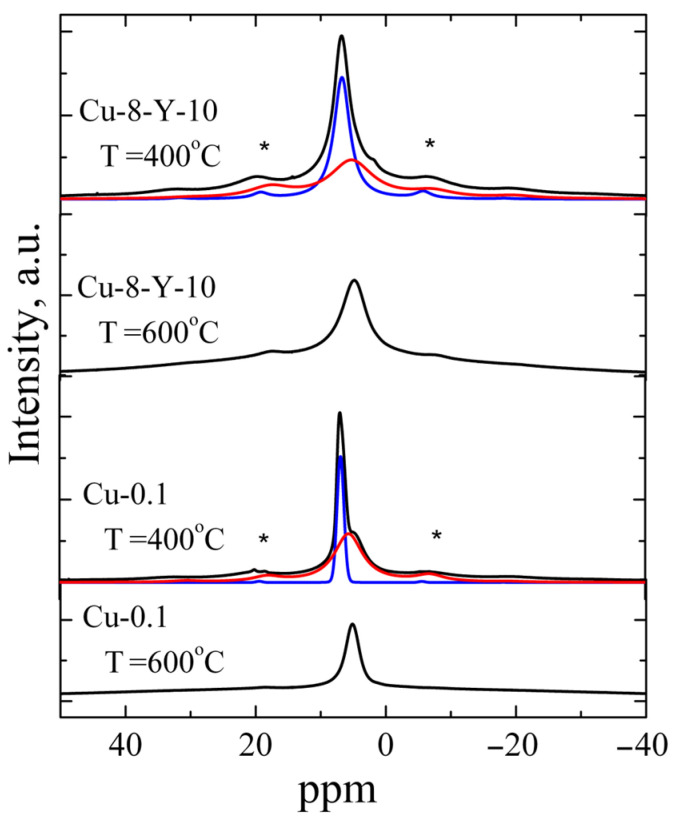
Experimental spectra ^1^H MAS NMR of ZrO_2_ powders doped with 0.1 mol.% CuO and ZrO_2_ powders co-doped with 8 mol.% CuO and 10 mol.% Y_2_O_3_ calcined at different temperatures (black curves) and also fitting components (red and blue curves). Asterisks mark the spinning sidebands.

Yttrium is frequently used as a stabilizing dopant for the tetragonal and cubic phases of zirconium oxide because it can remain within the ZrO_2_ host matrix up to 1400 °C [17]. Therefore, understanding the influence of yttrium incorporation on copper-related paramagnetic centers in ZrO_2_ materials is crucial. It is seen in Figure 3d–i that Cu-Y co-doping leads to the appearance of signal C at a lower calcination temperature, more specifically, at 800 °C, if compared to Cu-doped ZrO_2_ powders. For a series of samples with the same copper content, the maximum intensity of signal C was observed for samples doped with 3 mol.% of Y_2_O_3_. A possible explanation is that additional doping with yttrium, which has a diffusion coefficient several times larger than that of copper [25], accelerates the formation of oxygen vacancies required for dopant charge compensation. This, in turn, facilitates the simultaneous diffusion of copper into lattice sites at lower calcination temperatures. However, a further increase in the yttrium content up to 10 mol.% Y_2_O_3_, will lead to a competition between yttrium and copper in the occupation of cation sites because of the larger amount of Y ions. Consequently, the initial increase in the amount of Cu^2+^_Zr_ with yttrium doping changes to a decrease once the critical yttrium content is reached. This is due to a complex interplay of factors affecting the structure and stability of the lattice. Most authors of studies on copper-doped oxides attribute EPR signals similar to the above-mentioned signal A to surface complexes of copper with H_2_O or OH. To clarify this issue, the ^1^H MAS NMR technique was applied to the powders studied. As an example, ^1^H MAS NMR spectra of ZrO_2_ powders doped with 0.1 mol.% CuO (the lowest impurity loading) and ZrO_2_ co-doped with 8 mol.% CuO and 10 mol.% Y_2_O_3_ (the highest impurity loading) are shown in Figure 4.

All powders annealed at 400 °C were found to exhibit ^1^H MAS NMR signals consisting of two components: an intense band at δ = 6.83 ppm and a weak band at δ = 5.53 ppm. The components at δ = 5.53 ppm were observed to dominate in the powders calcined at 600 °C, while this ^1^H NMR signal was not detected in the samples calcined at higher temperatures (≥800 °C). Thus, the band at δ = 5.53 ppm was assigned to Zr-OH groups, while the band at 6.83 ppm is related to protons of H_2_O molecules on the oxide surface [26,27,28,29]. The line width of both components was found to be strongly influenced by the number of protons involved in forming hydrogen bonds and decreased with increasing annealing temperature. The reduction in the contribution of the component at δ = 5.53 ppm observed at higher annealing temperatures indicates the escape of water molecules at 600 °C. This finding correlates with previously obtained IR spectroscopy data on the yield of adsorbed water and hydroxyl groups in ZrO_2_ powders [30]. The escape of water molecules is accompanied by a sharp decrease in signal A in the EPR spectra, which confirms the assignment of the signal to surface Cu^2+^-(H_2_O)_x_ complexes.

**Figure 5:**
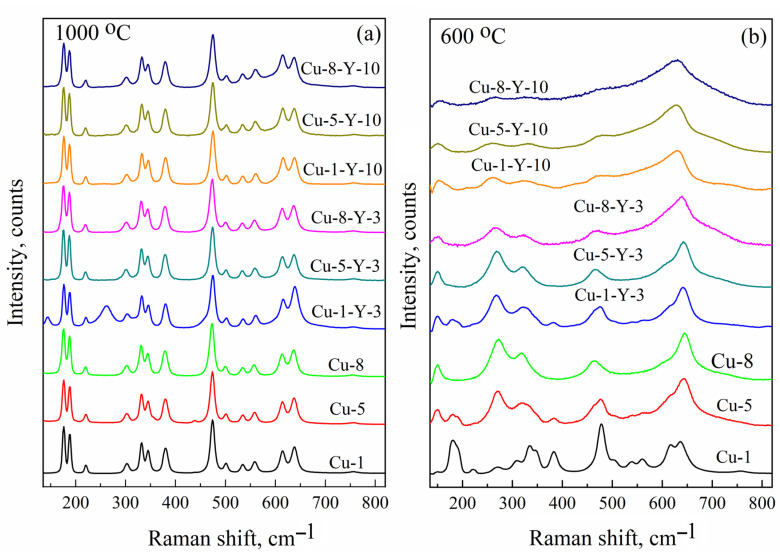
Raman shift spectra of Cu-doped and (Cu,Y)-co-doped ZrO_2_ powders calcined at 1000 (**a**) and 600 °C (**b**).

To confirm the model of the paramagnetic center responsible for the C signal, namely Cu_Zr_^2+^ cation positions in the monoclinic ZrO_2_, the same powders were studied using Raman shift spectroscopy. Figure 5 presents Raman spectra of Cu-doped and (Cu,Y)-co-doped ZrO_2_ powders calcined at 600 and 1000 °C. The number of phonon modes that appear in the Raman spectrum of ZrO_2_ is known to be governed by its crystalline structure [4,31,32]. Although the peak positions of some of them are nearly the same for different phases (for instance, the strong A_g_ mode at 476 and 475 cm^−1^ for monoclinic and tetragonal phases, respectively), there are strong phonon modes specific only to monoclinic (A_g_ doublet at 183 cm^−1^ and 193 cm^−1^), tetragonal (B_1g_ mode at 155 cm^−1^ and E_g_ mode at 257 cm^−1^), and cubic (E_2g_ mode at 607 cm^−1^) ZrO_2_ phases [31,32]. Thus, these phonon modes are considered fingerprints for each crystalline phase and can be used to identify their contribution to the powder structure. As shown in Figure 5a, all powders calcined at 1000 °C exhibit a characteristic A_g_ doublet at approximately 183 and 193 cm^−^¹, indicating the dominant contribution of the monoclinic ZrO_2_ phase. The corresponding EPR spectra show the C signal, with its intensity depending on the Cu and Y content, as seen in Figure 3. Indeed, for the Cu-1-Y-3 powder, its contribution is lower and aligns with the presence of the tetragonal phase (revealed by the B_1g_ and E_g_ modes) in the Raman spectra (Figure 5b). This finding corroborates the absence of the C signal in the EPR spectra of powders calcined at 600 °C (see Figure 3) and demonstrates the dominant contribution of tetragonal ZrO_2_ (see Figure 5b).

This correlation of EPR and Raman shift spectra supports the proposed model of the paramagnetic center responsible for the C signal, namely the Cu^2+^_Zr_ cation position in monoclinic ZrO_2_. Thus, the Cu-doped zirconium oxide powders studied in this work yielded three EPR signals that can be described using the following parameters: g_⊥_ = 2.063, g_||_ = 2.340, and A_||_ = 130 G (signal A), g ~ 2.17 (signal B), and g_x_ = 2.021, g_y_ = 2.026, g_z_ = 2.168, A_xx_ ~ 40 × 10^−4^ cm^−1^, A_yy_ ~ 35 × 10^−4^ cm^−1^, A_zz_ ~ 186 × 10^−4^ cm^−1^, A_xz_ ~ 10 × 10^−4^ cm^−1^, and A_yz_ ~ 5 × 10^−4^ cm^−1^ (signal C). They can be identified as surface Cu(H_2_O)_x_ complexes, superparamagnetic Cu and/or CuO_x_ nanoclusters at the grain boundaries, and Cu^2+^_Zr_ in monoclinic ZrO_2_, respectively. Identification of the EPR signals enables tracing the formation of copper-related paramagnetic centers in Cu-doped zirconium dioxide powders produced under different process parameters.

Signal A was found to dominate in powders calcined at low temperatures (400 °C) and with low dopant concentrations (≤1 mol.% CuO). This indicates that, under these synthesis conditions, the copper alloying impurity ions were located on the surface of the zirconium dioxide powder grains, interacted with absorbed water, and formed surface Cu^2+^-(H_2_O)_x_ complexes. At higher concentrations (5 and 8 mol.% CuO), the dopant atoms interacted with each other, forming superparamagnetic Cu or/and CuO_x_ nanoclusters on the grain boundaries. When the calcination temperature was elevated to 600 °C, signal B became dominant in the EPR spectrum of Cu-doped ZrO_2_ powders. This implies the destruction of surface Cu^2+^-(H_2_O)_x_ complexes at this temperature, which was confirmed by ^1^H NMR data on the release of adsorbed water. The copper ions liberated during this process drifted along the granule surface, beginning to interact with each other and forming superparamagnetic clusters on the grain boundaries. Increasing the calcination temperature to 1000 °C was observed to lead to the destruction of superparamagnetic clusters and the incorporation of copper ions into the zirconium dioxide structure as Cu^2+^_Zr_ cationic substitutions.

## 4. Conclusions

In this study, the comparative study of Cu-doped and (Cu,Y)-codoped ZrO_2_ nanopowders prepared by the co-precipitation approach and calcined at different temperatures was performed by means of EPR, NMR, and Raman scattering methods. As a result, the presence of three signals in the EPR spectra of the samples was found as follows: (i) signal A with g_⊥_ = 2.063, g_||_ = 2.340, and A_||_ = 130 G; (ii) signal B with g ~ 2.17, and (iii) signal C with g_x_ = 2.021, g_y_ = 2.026, g_z_ = 2.168, A_xx_ ~ 40 × 10^−4^ cm^−1^, A_yy_ ~ 35 × 10^−4^ cm^−1^, A_zz_ ~ 186 × 10^−4^ cm^−1^, A_xz_ ~ 10 × 10^−4^ cm^−1^, A_yz_ ~ 5 × 10^−4^ cm^−1^. Based on a comparison of the results obtained by EPR, ^1^H NMR, and Raman methods, these signals were attributed to the surface Cu^2+^-(H_2_O)_x_ complexes, superparamagnetic Cu or/and CuO_x_ nanoclusters on the grain boundaries, and Cu^2+^_Zr_ cations in the monoclinic ZrO_2_, respectively. The contribution of these signals to the analyzed EPR and NMR spectra was found to depend on dopant concentrations and calcination temperature. The surface Cu^2+^-(H_2_O)_x_ complexes were concluded to form in the doped ZrO_2_ powders with low dopant concentrations (≤1 mol.% CuO) at low temperatures (400 °C). Superparamagnetic nanoclusters were observed in powders calcined at temperatures of 600 and 800 °C. An increase in the Cu concentration led to a decrease in the temperature of the formation of nanoclusters; that is, at concentrations larger than 1 mol.% CuO, they formed at 400 °C. High calcination temperature (1000 °C) resulted in the incorporation of copper ions into the zirconium dioxide lattice as Cu^2+^_Zr_ substitution ions.

Thus, a comprehensive systematic study of copper-doped zirconium dioxide powders revealed that the incorporation of copper into the ZrO_2_ powder structure is influenced by both the concentration of the alloying impurity and the calcination temperature. Varying the calcination temperature and the levels of yttrium and copper doping can effectively control the surface state of composite nanoparticles and the formation of copper complexes formed there. This, in turn, allows for the tuning and enhancement of the material’s catalytic properties. Moreover, the use of the EPR method and the identification of copper-related paramagnetic centers have proven to be powerful tools in detecting small quantities of paramagnetic centers and studying the formation and localization of copper complexes in Cu-doped ZrO_2_ powders.

## Figures and Tables

**Table 1:** Composition of powders prepared and analyzed in the present study.

Powder Title	ZrO_2_ Content, mol.%	CuO Content, mol.%	Y_2_O_3_ Content, mol.%
Cu-0.1	99.9	0.1	-
Cu-1	99.0	1.0	-
Cu-5	95.0	5.0	-
Cu-8	92.0	8.0	-
Cu-1-Y-3	96.0	1.0	3.0
Cu-5-Y-3	92.0	5.0	3.0
Cu-8-Y-3	89.0	8.0	3.0

## Data Availability

The original contributions presented in the study are included in the article, further inquiries can be directed to the corresponding author.

## Abbreviations

The following abbreviations are used in this manuscript:

EPR	Electron paramagnetic resonance
NMR	Nuclear magnetic resonance
